# Sleep quality and the cortisol and alpha-amylase awakening responses in adolescents with depressive disorders

**DOI:** 10.1192/bjo.2024.730

**Published:** 2024-08-06

**Authors:** Rebekka Krempel, Irina Jarvers, Angelika Ecker, Daniel Schleicher, Romuald Brunner, Stephanie Kandsperger

**Affiliations:** Department of Child and Adolescent Psychiatry, Psychosomatics and Psychotherapy, University of Regensburg, Germany

**Keywords:** Depressive disorders, sleep, adolescence, cortisol, amylase

## Abstract

**Background:**

Depressive disorders in adolescents affect all aspects of life and impose a very large burden of disease. Sleep is frequently affected by depression and is crucial for facing challenges during development. One of the postulated reasons for depression-induced sleep disruption is dysregulation of the physiological stress system.

**Aims:**

To investigate the links of adolescent depressive disorders with subjective sleep quality, objective sleep quality, and the course of cortisol and alpha-amylase after awakening.

**Method:**

We compared subjective sleep quality (via daily questionnaires) and objective sleep quality (via actigraphy measurement) of 35 adolescents with depressive disorders and 29 healthy controls over 7 consecutive days. In addition, saliva samples were collected on 3 days to examine cortisol and alpha-amylase patterns after awakening.

**Results:**

No significant differences in cortisol or alpha-amylase awakening responses were observed between participants with depressive disorders and healthy controls. We found severe reductions in subjective sleep quality in the depression group (*Z* = −5.19, *P* < 0.001, *d* = 1.80) and a prolonged actigraphy-measured sleep onset latency (*Z* = −2.42, *P* = 0.015, *d* = 0.64) compared with controls. Reductions in subjective sleep quality were partially correlated with objective sleep measures (sleep onset latency: *r* = −0.270, *P* = 0.004, sleep efficiency: *r* = 0.215, *P* = 0.017).

**Conclusions:**

Sleep onset latency seems to aggravate depressive symptoms and to have an important role in perception of sleep quality. Adolescents with depressive disorders should be supported regarding the establishment of good sleep hygiene and avoiding activities that may impede falling asleep.

## Adolescent depression and sleep quality

Depressive disorders are common during adolescence, with a point prevalence of approximately 8%^1^ and a considerable increase in onset risk through adolescence.^2^ Reduced sleep quality is a widely experienced symptom accompanying major depressive disorder (MDD). Previous research has indicated that sleep problems accompanying depression may be even more frequent in adolescents than in adults.^3^

Sleep quality can be subdivided into objective and subjective aspects. Objective aspects comprise sleep onset latency, sleep duration, sleep efficiency, sleep stages and sleep patterns and can be measured by polysomnography or actigraphy. Subjective characteristics include estimated sleep onset latency, estimated frequency of nocturnal awakening, quality of dreams, presence of unintended early awakening and restfulness of sleep. These aspects are usually assessed via questionnaires. Consequences of impaired sleep quality include higher severity of current^4^ and later depressive symptoms,^5^ increased anxiety^6^ and decreased positive emotions.^7^ A previous study in a nonclinical adolescent sample found a positive correlation between later sleep onset and depressive symptom severity and a negative correlation between total sleep duration and depressive symptom severity.^8^ Moreover, sleep problems increase the risk of dying by suicide in adolescence.^9^

## Relationship between sleep and biomarkers of stress systems

Sleep has a modulating effect on the hypothalamic–pituitary–adrenal (HPA) axis.^10^ The HPA axis is also known in particular for its role in the stress response, which is primarily mediated by the stress hormone cortisol.^11^ Dysregulation of the HPA axis and subsequent alterations in cortisol secretion comprise a common hypothesis in neuroendocrinological research on depressive disorders. Cortisol secretion follows a characteristic diurnal cycle with an early morning peak, a prompt increase after awakening (cortisol awakening response (CAR)) and a decline throughout the day.^12^ Regarding adolescent depression, previous research is heterogeneous, with some studies observing an increase, some finding a decrease and others identifying no difference in levels of morning cortisol in adolescent participants with depressive disorders.^13^

Another biomarker besides cortisol that plays an important part in the stress response is salivary alpha-amylase.^14^ Many studies also indicate the relevance of salivary alpha-amylase in the context of sleep.^15^ The diurnal secretion pattern of alpha-amylase in adolescents is not well understood; some studies have reported a characteristic decrease within the first 30 min after awakening (amylase awakening response (AAR)^16^), whereas other research did not observe a decrease.^17^ During the day, amylase levels increase.^16,17^ However, secretion patterns and levels seem to be variable and depend on the stage of puberty.^17^

In a sample of adults with depression, Bauduin et al found elevated levels of alpha-amylase after awakening compared with healthy controls and compared with individuals with other psychiatric disorders; they proposed alpha-amylase as a ‘new candidate biomarker for MDD specifically’.^18^ However, research concerning alpha-amylase secretion, especially in adolescents with depressive disorders, has been very limited.^19^ In a sample of adolescents with depression, Jezova et al found no differences in activity of morning alpha-amylase, lower midday alpha-amylase activity, and no increase in enzyme activity throughout the day compared with healthy controls.^19^

## Hypotheses

We predicted that adolescents with depressive disorders would have lower objective and subjective sleep quality than healthy controls. We also expected significant differences in CAR and AAR in the depression group compared with healthy controls.

## Method

### Study design

This non-interventional, single-centre study was conducted at the Department of Child and Adolescent Psychiatry, Psychosomatics and Psychotherapy at the University of Regensburg, Germany. The study protocol has been previously published.^20^

### Sample

Several *a priori* power analyses were conducted with the program G*Power^21^ depending on the statistical method. Determined sample sizes were *n* = 28 for repeated measures, *n* = 44 for correlations and *n* = 60 for direction-dependence analysis (see ref. ^20^ for details).

We recruited participants for the depression group from the out-patient and day clinic to minimise sleep alterations by measuring sleep in a familiar environment. Patients with diagnosed single-episode and recurrent MDD (F32.0, F32.1, F32.2, F33.0, F33.1, F33.2) or adjustment disorder with prolonged depressive reaction (F43.21) according to the ICD-10 diagnostic criteria^22^ without psychiatric medication were eligible for participation. Intake of antidepressants could be a factor influencing the inconsistency of the results; hence, we only included participants who were not taking antidepressants.^23^ Except for anxiety disorders, no comorbid psychiatric disorders were included. Furthermore, the exclusion criteria were psychotic or acute suicidal conditions, cannabis consumption within 3 months before participation, pregnancy, neurological or endocrinological illnesses with a known influence on brain or sleep, and intake of glucocorticoid medication.

The control group was recruited via advertisement in mailing lists, social media channels, websites of the study hospital, and via a local TV channel. Adolescent participants without current or past psychiatric diseases or a history of psychotherapeutic or psychiatric intervention were age-matched to those in the depression group. The same exclusion criteria were applied as in the patient group. We included 35 adolescents in the depression group and 29 adolescents in the control group after excluding one adolescent reporting a prior depressive episode.

### Ethics and consent

Written informed consent from participants and their legal guardians was required for participation and was obtained before any assessment was conducted. Withdrawal by adolescents or their legal guardians was possible at any time.

The authors assert that all procedures contributing to this work comply with the ethical standards of the relevant national and institutional committees on human experimentation and with the Helsinki Declaration of 1975, as revised in 2008. All procedures involving human patients were approved by the Ethics Committee of the University of Regensburg (reference number 20-1711-101).

As an expense allowance, all participants received a €50 gift voucher.

### Assessments

#### Measurements on the first study day

Psychiatric diagnoses were conducted by qualified clinicians under the supervision of a certified child and adolescent psychiatrist using the Mini-International Neuropsychiatric Interview for Children and Adolescents 6.0^24^ according to the DSM-IV and ICD-10 criteria. We assessed depressive symptoms in the past 2 weeks with the Beck Depression Inventory-II (BDI-II)^25^ but excluded item 21 (loss of interest in sexuality) to adapt this questionnaire to the younger participants. Somatic anxiety symptoms in the past 2 weeks were evaluated with the Beck Anxiety Inventory (BAI).^26^

Sleep quality in the past 2 weeks was evaluated with the Sleep Questionnaire B (SF-B/R).^27^ Sleeping traits (chronotype, regularity of bedtime, duration of sleep needed to feel rested, and frequency of sleeping through school or work) were assessed with a self-designed sleeping trait questionnaire. To assess stress vulnerability and the use of stress coping strategies, we administered the Stress and Coping Questionnaire for Children and Adolescents (SSKJ 3-8 R).^28^ Pubertal status was evaluated with the self-report Pubertal Development Scale (PDS).^29^

For a detailed description of the questionnaires used, please see Supplementary Table 1 available at https://doi.org/10.1192/bjo.2024.730.

#### Sleep questionnaires during measurement week

For 7 consecutive days (measurement week), participants reported their satisfaction with the past night's sleep, current mood and feeling of recreation, difficulties with sleeping in, nocturnal awakening, stress level in the past day, presence of nightmares, quantity of caffeinated beverages consumed, quantity of alcoholic beverages consumed, and sleeping and wake-up times on a self-designed sleep questionnaire completed in the morning after awakening.

#### Accelerometry

During the measurement week (Monday to Sunday), participants wore an Act Trust 2 wrist actimeter (Condor Instruments) on their non-dominant hand overnight for 7 consecutive nights. The device recorded data in 1-min intervals during the night. Participants were instructed to mark their intended sleeping time, any occurrences during the night (for example, going to the bathroom) and their wake-up time by pushing the event button on the device.

Recordings were checked, and the main sleeping periods were identified with ActStudio software (Condor Instruments, São Paulo, Brazil, PC version 1.0.10, for Windows). Bedtime, wake-up time, time in bed, total sleep time, sleep onset latency, sleep efficiency, wakefulness after sleep onset and awakenings were derived for each night and automatically averaged for subsequent analysis.

#### Saliva sampling

On three consecutive mornings during the measurement week (Tuesday to Thursday), participants provided saliva samples directly after awakening (baseline), 30 min after awakening (CAR peak) and 45 min after awakening (CAR decline). According to Zajkowska et al,^30^ the assessment of CAR has been quite heterogeneous throughout studies examining CAR in adolescents with depressive disorder; therefore, we orientated our CAR assessment to the expert consensus guidelines for CAR.^31^ To simplify handling for the participants, salivettes were labelled with sampling times and marked with different colours for each sampling day. No eating, drinking or tooth brushing was allowed 10 min before sampling, and accurate sampling was demonstrated and practised on the first study day. In addition, participants were provided with written instructions for saliva sampling. Salivettes (Salivette Cortisol, Sarstedt, Nümbrecht, Germany, item number 51.1534.500) were used and stored in a container equipped with the Medication Event Monitoring System (MEMS^®^, Aardex, Liege, Belgium) to check sampling times. We informed the participants about the monitoring before sampling to improve the accuracy of sampling times. After sampling, we asked participants to store the samples in the freezer. On the second study day, samples were stored at −20 °C, and cortisol and alpha-amylase levels were determined by the Department of Biopsychology, Technical University of Dresden, Germany. See Supplementary Table 2 for an overview of sampling times, and refer also to the supplementary information for details of the alpha amylase assay analysis.

#### Measurement consistency

For all participants, the measurement week began on a Sunday evening and included the same number of weekdays and weekend days. To control for the influence of the menstrual cycle on the CAR,^32^ female participants not taking contraception participated during their luteal phase.

### Statistical analyses

In the first step, group differences in control variables were examined via *t*-tests, equivalence tests and Mann‒Whitney *U*-tests. To examine differences in cortisol and alpha-amylase measurements over time, values were square-root transformed to achieve a normal distribution^33^ and averaged over the 3 days. Repeated-measures analysis of covariance (ANCOVA) was performed with cortisol and alpha-amylase levels as dependent variables and group, PDS scores, BDI scores and BAI scores as independent variables. As a further measure, the cortisol increase and alpha-amylase decrease were computed via area under the curve analyses relative to ground/increase (AUCg/AUCi)^34^ and delta scores between the baseline value and the peak value.^35,36^ Regarding subjective and objective sleep quality, group differences were computed depending on variable distribution. Kendall's τ correlations were used to examine the relationship between objective and subjective measures. A false discovery rate correction was applied where appropriate.^37^ Effect sizes are reported with Cohen's *d*, with 0.2 corresponding to a small effect, 0.5 to a medium effect and 0.8 to a large effect. As a final step, direction dependence analysis (DDA) was used to examine the direction of the relationship between sleep quality and depressive symptoms^38^ while controlling for pubertal status as a covariate. This was possible because the main variables were not normally distributed. All statistical analyses were conducted using IBM SPSS version 29.0 for Windows with a *P*-value threshold of 0.05 for significance. For additional details, see the research protocol.^20^

## Results

The demographic and clinical characteristics of our study sample are shown in Table 1. There was statistical equivalence in age between the groups, but the depression group contained a higher proportion of female participants. In the depression group, pubertal development was more advanced compared with that of healthy controls, whereas the control group had a higher level of secondary school than the depression group. Most participants in the depression group had a first, moderate depressive episode (71.4%). In addition, 37.1% of the depression group was diagnosed with comorbid social phobia (F40.1). As expected, the groups differed strongly in mean BDI-II and BAI scores but not in use of hormonal contraceptives (Table 1).

**Table 1:** Sample characteristics

	Depression group	Healthy control group	Group differences
Sample size	35 (100%)	29 (100%)	
Sex			*t*(55) = −0.69, *P* = 0.245^a^
Female	28 (80%)	19 (65.5%)	
Male	7 (20%)	10 (34.5%)	
Mean age in years (s.d.)	15.20 (1.69)	15.00 (1.69)	*t*(60)=−1.88, *P* = 0.032^a^
Pubertal status			*Z* = −2.13, *P* = 0.033^b^
Prepubertal	1 (2.9%)	–	
Early pubertal	–	3 (10.3%)	
Mid-pubertal	3 (8.6%)	7 (24.1%)	
Late pubertal	19 (54.3%)	13 (44.8%)	
Post-pubertal	12 (34.3%)	6 (20.7%)	
School type^c^			*Z* = −2.90, *P* = 0.004^b^
Gymnasium	13 (37.1%)	19 (65.5%)	
Fachoberschule/Berufsoberschule	1 (2.9%)	2 (6.9%)	
Realschule	9 (25.7%)	7 (24.1%)	
Mittelschule	12 (34.3%)	1 (3.4%)	
Depression diagnosis (ICD-10)			
F32.0	5 (14.3%)	–	
F32.1	25 (71.4%)	–	
F32.2	1 (2.9%)	–	
F33.0	1 (2.9%)	–	
F33.1	2 (5.7%)	–	
F33.2	1 (2.9%)	–	
Comorbid anxiety disorder			
F40.1	13 (37.1%)	–	
BDI mean (s.d.)	23.20 (13.87)	2.17 (3.59)	*Z* = −6.28, *P* < 0.01^b^
BAI mean (s.d.)	19.51 (13.60)	3.72 (5.51)	*Z* = −5.30, *P* < 0.01^b^
Hormonal contraceptive intake (female)	3 (8.6%)	3 (10.3%)	χ^2^(1) = 0.26, *P* = 0.609^d^

BDI, Beck Depression Inventory II (possible values from 0 to 60 owing to exclusion of item 21); BAI, Beck Anxiety Inventory (possible values from 0 to 63); F32.0, mild depressive episode; F32.1, moderate depressive episode; F32.2, severe depressive episode without psychotic symptoms; F33.0, recurrent depressive disorder, current episode mild; F33.1, recurrent depressive disorder, current episode moderate; F33.2, recurrent depressive disorder, current episode severe without psychotic symptoms.

a. Equivalence test.

b. Mann–Whitney *U*-test.

c. School types refer to secondary schools following elementary school in Germany. Gymnasium: highest level of secondary school, regular duration of 8–9 years, general qualification for university entrance; Fachoberschule/Berufsoberschule: tertiary school to achieve advanced technical college certificate, subject-related entrance qualification or general qualification for university entrance after visiting Realschule, duration of 2–3 years in addition to duration of Realschule; Realschule: intermediate level of secondary school, regular duration of 6 years; Mittelschule: 9 years of elementary school.

d. Chi-squared test.

### Objective sleep quality

A significant group difference was found in sleep onset latency (*Z* = −2.423, *P* = 0.015). Other objective sleep quality variables (total sleep time, sleep efficiency, wakefulness after sleep onset, awakenings) did not differ significantly between the two groups (Table 2).

**Table 2:** Group differences in cortisol increase, alpha-amylase decrease, subjective sleep quality and objective sleep quality

		Depression group	Healthy control group	Group differences	
		Mean (s.d.)	Mean (s.d.)	*Z*	*P*-value
Biomarkers	ΔCORT increase (nmol/L)	9.72 (7.48)	8.57 (5.79)	−0.28	0.782
	ΔAMYL decrease (U/mL)	39.50 (43.53)	36.18 (35.12)	−0.64	0.522
Subjective sleep quality^a^	Daily sleep quality questionnaire	19.26 (3.11)	23.79 (1.95)	−5.20	<0.001
	Total sleep time	8.24 (2.26)	8.18 (1.40)	−0.76	0.446
	Difficulty falling asleep	3.33 (1.21)	2.81 (1.24)	−3.01	0.003
	Difficulty staying asleep	2.52 (0.92)	1.63 (0.50)	−4.10	0.001
	Premature awakening	1.93 (1.72)	1.44 (1.28)	−2.03	0.042
	Sleep quality	3.10 (0.77)	4.19 (0.49)	−5.05	<0.001
Objective sleep quality^b^	Total sleep time, h	7:04 (0.59)	7:20 (0:53)	−0.86	0.389
	Onset latency, h	0:16 (0.17)	0:08 (0:06)	−2.42	0.015
	Sleep efficiency, %	88.59 (6.87)	90.95 (4.77)	1.28	0.200
	Wakefulness after sleep onset, h	0:31 (0.19)	0:30 (0:19)	−0.29	0.772
	Awakenings	7.20 (3.63)	7.93 (4.26)	−0.56	0.576

CORT, cortisol; AMYL, alpha-amylase.

a. Subjective sleep quality: total sleep time, difficulty falling asleep, difficulty staying asleep, premature awakening (sleep indices of Sleep Questionnaire B); sleep quality: factor scale of Sleep Questionnaire B.

b. Objective sleep quality: actigraphy-measured variables, analyses of group differences with Mann–Whitney *U*-test.

### Subjective sleep quality

Group comparisons regarding subjective and objective sleep quality are shown in Table 2. Significant group differences were found in the mean score on the daily sleep questionnaire (*Z* = −5.19, *P* < 0.001), sleep index for difficulties falling asleep (*Z* = −3.01, *P* = 0.003), sleep index for staying asleep (*Z* = −4.103, *P* < 0.001), sleep index for premature awakening (*Z* = −2.03, *P* = 0.042) and factor scale for sleep quality (*Z* = −5.045, *p* < 0.001). Scores on our custom-designed daily sleep questionnaire were correlated strongly with the factor scale sleep quality of the well-established sleep questionnaire (SF-B/R) (*r* = 0.51, *P* < 0.001).

### Correlations of subjective and objective sleep quality with depressive symptoms

Correlations of subjective and objective sleep quality parameters with the severity of depressive symptoms are shown in Table 3. There was a significant correlation between sleep quality as reported on the daily sleep questionnaire and sleep onset latency (*r* = −0.27, *P* = 0.004) as well as sleep efficiency (*r* = 0.22, *P* = 0.017). The subjective estimation of total sleep time was significantly correlated with the actigraphy-measured objective total sleep time (*r* = 0.30, *P* = 0.001). Moreover, subjective estimation of sleep onset difficulties (SF-B/R sleep index: difficulty falling asleep) and actigraphy-measured sleep onset latency were significantly correlated (*r* = 0.22, *P* = 0.021). Regarding difficulties in sleeping through the night, there was no correlation between subjective estimation and actigraphy-measured variables (awakening, wakefulness after sleep onset).

**Table 3:** Correlations between subjective sleep quality, objective sleep quality and depressive symptoms (total sample)

		Objective sleep quality^a^					BDI depression symptom severity^c^
		Total sleep time	Sleep onset latency	Sleep efficiency	Wakefulness after sleep onset	Awakenings	
Subjective sleep quality^b^	Daily sleep quality questionnaire	0.03	−0.27**	0.22*	−0.11	−0.03	−0.52***
	Total sleep time	0.30**	0.16	−0.03	0.06	0.09	−0.08
	Difficulty falling asleep	−0.39	0.22*	−0.10	0.05	0.11	0.35***
	Difficulty staying asleep	−0.09	0.12	−0.16	0.14	0.10	0.48***
	Premature awakening	−0.07	0.00	−0.08	0.04	−0.03	0.26*
	Sleep quality	0.08	−0.15	0.10	−0.08	−0.08	−0.53***
BDI	Depression symptom severity	−0.19*	0.22*	−0.16	0.10	−0.02	−

a. Objective sleep quality: actigraphy-measured variables.

b. Subjective sleep quality: total sleep time, difficulty falling asleep, difficulty staying asleep, premature awakening (sleep indices of Sleep Questionnaire B); sleep quality: factor scale of Sleep Questionnaire B.

c.Depression symptom severity according to mean Beck Depression Inventory (BDI) II score.

*P < 0.05, **P < 0.01, ***P < 0.001.

Depressive symptoms were correlated with sleep quality as reported on the daily questionnaire (*r* = −0.52, *P* < 0.001) and the SF-B/R factor scale for sleep quality (*r* = −0.53, *P* < 0.001). Moreover, depressive symptoms were correlated with the sleep indices for difficulties falling asleep (*r* = 0.35, *P* < 0.001), staying asleep (*r* = 0.48, *P* < 0.001) and premature awakening (*r* = −0.26, *P* = 0.010). Regarding objective sleep quality parameters, depressive symptoms were correlated with actigraphy-measured total sleep time (*r* = −0.19, *P* = 0.033) and sleep onset latency (*r* = 0.22, *P* = 0.015).

### Direction dependence analysis

DDA was used to evaluate two competing regression models of depression/anxiety symptoms (objective sleep onset latency → depression/anxiety symptoms (BDI/BAI scores) versus depression/anxiety symptoms (BDI/BAI scores) → objective sleep onset latency) while adjusting for the covariate of PDS. The first model was favoured by the skewness and kurtosis tests (*z* = 2.02, *P* = 0.043; *z* = 2.77, *P* = 006), homoscedasticity tests (χ^2^ = 22.10, *P* < 0.001) and nonlinear correlation tests using the square function (*t*(61) = 2.44, *P* = 0.018), suggesting that sleep onset latency causally influenced depression and anxiety symptoms. Regarding subjective sleep, DDA was not able to clearly decipher the causal direction.

### Cortisol and alpha-amylase

**Fig. 1:**
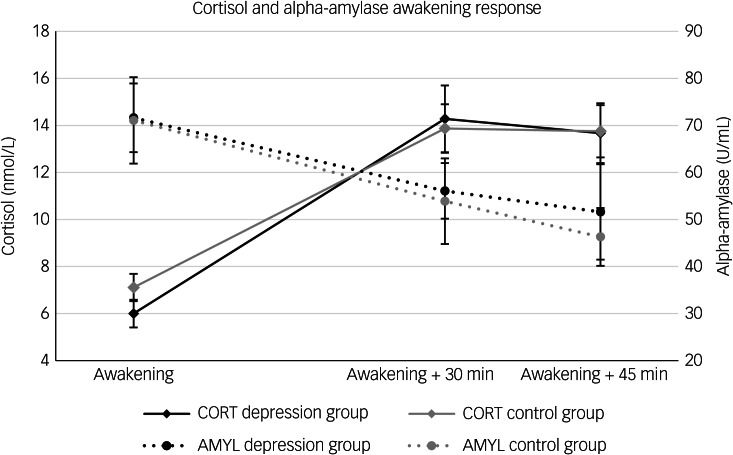
Cortisol and alpha-amylase awakening response of depression group and healthy control group. CORT, cortisol; AMYL alpha-amylase.

The averaged courses of cortisol and alpha-amylase after awakening are presented in Fig. 1. There was no significant group difference regarding the time course of cortisol (*F*(2.59) = 0.39, *P* = 0.628). In addition, no significant group difference was found for the time course of alpha-amylase (*F*(2.59) = 1.41, *P* = 0.248). Regarding control variables, there was a statistically significant effect of the interaction between time and pubertal status on cortisol (*F*(2.59) = 4.99, *P* = 0.014) and on alpha-amylase (*F*(2.59) = 3.86, *P* = 0.029) (see ANCOVA results in Supplementary Table 3). The results did not change when the BDI was included as a dimensional measure, nor when *n* = 2 individuals and *n* = 5 values per day were excluded according to the MEMS^®^ system.

No significant group differences were found for the AUCg values for cortisol (*Z* = −0.49, *P* = 0.623) or alpha-amylase (*Z* = −0.47, *P* = 0.642) levels, AUCi values for cortisol (*Z* = −0.59, *P* = 0.557) or alpha-amylase (*Z* = −0.05, *P* = 0.962), delta increase in cortisol (*Z* = −0.28, *P* = 0.782) or delta decrease in alpha-amylase (*Z* = −0.64, *P* = 0.522).

## Discussion

The present study assessed the sleep quality of adolescents with depressive disorders from different perspectives. The depression group showed significantly worse sleep quality and prolonged sleep onset latency compared with controls. However, the groups did not differ significantly in the magnitude of their CAR and AAR.

### Objective sleep quality

Our findings revealed prolonged sleep onset latency in the depression group but no group differences in sleep efficiency, wakefulness after sleep onset or number of awakenings. These results seem largely consistent with previous findings of sleep research in adolescents with depression, according to a review by Rao.^39^ Nine of 13 studies found no difference in sleep efficiency for the depression group, whereas three studies observed a decrease in sleep efficiency in this group. Our results are in accordance with most of these studies. Regarding sleep onset latency, seven studies showed no difference and five studies indicated prolonged sleep onset latency in the depression group; the latter is in line with the results of the present study. Regarding the prolonged sleep onset latency, our results align with a review by Ivanenko et al, who concluded that prolonged sleep onset latency is ‘the most consistent change in subjective and objective (instrumental) measurements of sleep’^40^ in young people with depressive disorders. The negative correlation between sleep duration and depressive symptoms found in the present study aligns with the findings of Thorburn-Winsor et al.^8^

### Subjective sleep quality

The results of our study underline the frequency and variety of subjective sleep quality reductions in adolescents with depressive disorders. Adolescents with depressive disorders reported lower overall sleep quality but also problems in all stages of sleep. Our findings confirm the longer subjective sleep onset latency and longer subjective wakefulness after sleep onset previously observed in adolescents with depression.^41^ However, Sivertsen et al also found a decrease in subjective total sleep time in adolescents with depression,^41^ which was not confirmed in our study. In our study, reductions in sleep quality coincided in different measurements that assessed sleep quality in the previous 2 weeks as well as daily sleep quality.

Several participants in the depression group indicated that they were unable to complete the questionnaires in the morning because of bad mood and apathy. This raises the question of whether an assessment including an essential feature in the morning is suitable and feasible for use in a cohort of participants with depressive disorders, as these individuals are likely to experience pronounced morning lows. However, we are convinced that assessments such as ours help to visualise all aspects of the burden of disease. This is emphasised by the ability of the majority of participants to complete the morning assessment, although they were burdened or had experienced unsatisfying sleep.

### Relationship between subjective and objective sleep quality

In our study, subjective sleep onset latency was prolonged in the depression group, but the objective wake time after sleep onset was not increased, and the number of awakenings was not significantly higher compared with the control group. Nonetheless, these aspects were rated significantly worse in the questionnaires by the depression group. This may indicate that during retrospective assessment the next morning, prolonged sleep onset latency may be generalised to some extent and may negatively affect the overall perception of sleep. Thus, sleep onset latency may be an interesting target for treating sleep problems in adolescents with depression, because reducing it could improve perceived sleep quality noticeably.

However, the reduction in subjective sleep quality in our study was correlated with decreased objective sleep efficiency. Similarly, subjective problems falling asleep were correlated with increased objective sleep onset latency, and estimated and objectively measured total sleep time were correlated. These findings partially contrast with those of Rao, who assumed a mismatch between subjective and objective sleep quality in her review,^34^ and those of Thorburn-Winsor et al, who found a mismatch between estimated and actigraphy-measured total sleep time.^8^

### Cortisol and alpha-amylase

We found no significant difference in cortisol or alpha-amylase levels after awakening between the depression group and the control group. Our results raise the question of whether biological correlates could be less decisive than sleep characteristics and cognitive assessments when trying to approach the issue of impaired sleep quality in adolescent depressive disorders. Similarly, a recent meta-analysis reported divergent findings regarding morning cortisol levels in depressed adolescents, with no overall difference in morning cortisol.^13^ As a biomarker for MDD in adolescents, cortisol may be useful early on but less meaningful over time.^13^ However, other findings have indicated an increase in CAR in a sample of female adolescents with depressive disorders.^42^ Our study included both males and females; however, given the higher proportion of female participants in both of our study groups, larger deviations in the CAR of female participants should have also been shown in our cohort.

There have been few studies of alterations in alpha-amylase levels after awakening in adolescents with depressive disorders. Our finding of no differences in morning alpha-amylase levels of adolescents with depression is in line with those of Jezova et al, who studied a sample with a similar mean age.^19^ By contrast, previous studies have found elevated levels of alpha-amylase at awakening, as well as elevated AUC values for morning alpha-amylase in adults with depressive disorders, and have even identified alpha-amylase as a ‘valuable candidate biomarker’ with high specificity for depressive disorders.^18^ Considering the dependence on development^43^ and pubertal status,^17^ interrelations could be less clear during adolescence; thus, alpha-amylase levels may be a better biomarker of depression in adults.

### Limitations

Some limitations of the study are important to note. Actigraphy measurements are less wide-ranging than polysomnography measurements and are unable to measure some important aspects (REM/non-REM). In addition, although we provided extensive verbal and written instructions to ensure the ecological validity of saliva sampling, some influencing factors in the home environment could not be controlled. Regarding methodology, the use of absorbent materials for saliva collection for alpha-amylase has been criticised by some authors.^44^ Although it would probably be better to collect passive drool samples here,^44^ results argue against a great influence. Possible differences should be the subject of further investigations. In addition, the generalisability of these findings may be limited owing to the exclusion of adolescents in in-patient settings, those taking antidepressant medication and those with other comorbidities besides anxiety disorders. Moreover, our sample size was limited, and the two groups differed in terms of sex distribution, pubertal status and educational attainment.

### Implications and future directions

Our findings highlight the importance of incorporating sleep quality in the diagnosis and treatment process of depressive disorders as early as possible and then continuously throughout the course of disease. As prolonged sleep onset latency seems to aggravate depressive symptoms and may play an important part in perception of sleep quality, adolescents with depressive disorders should be supported regarding the establishment of good sleep hygiene and avoidance of activities that may impede falling asleep. Psychoeducation, consistent evening routines and relaxation techniques could potentially be used to support adolescents with sleep onset problems and reduce suffering due to impaired sleep if they are regularly incorporated and practised in therapeutic care.

Perceived sleep quality seems to be a good indicator of some aspects of objective sleep quality; thus, a self-report assessment of important aspects of sleep quality should be integrated into the early assessment of adolescents showing symptoms of depressive disorder. In addition, adolescents with persistent difficulties falling asleep should be monitored for mental health problems such as depressive symptoms or pathological anxiety.

## Supporting information

Krempel et al. supplementary material 1 Krempel et al. supplementary material

Krempel et al. supplementary material 2 Krempel et al. supplementary material

Krempel et al. supplementary material 3 Krempel et al. supplementary material

Krempel et al. supplementary material 4 Krempel et al. supplementary material

## Data Availability

The data that support the findings of this study are available from the corresponding author, S.K., on reasonable request.
