# Supplementary material for: Sleep quality and the cortisol and alpha-amylase awakening responses in adolescents with depressive disorders
Source: BJPsych Open. 2024 Aug 6;10(5):e140. doi: 10.1192/bjo.2024.730 (PMC11698168; doi:10.1192/bjo.2024.730)
Supplement: Krempel et al. supplementary material 1 — Krempel et al. supplementary material [file S2056472424007300sup001.docx]

**Suppl. Table 1**

Measurements, study adaptions, and psychometric properties

| Questionnaire | Measured item | Adaptions in study design | Psychometric properties |
| --- | --- | --- | --- |
| **Measurements of the first study date** | | | |
| Mini-International Neuropsychiatric Interview for Children and Adolescents (M.I.N.I. KID 6.0) (1) | Potential symptoms of psychiatric disorders according to the DSM-IV and ICD-10 | Reported symptoms are doublechecked and, if necessary, corrected by an experienced specialist for child and youth psychiatry | positive predictive values heterogeneous, high negative predictive values (AUC ≥ .91), good interrater reliability (AUC ≥ .89), and retest reliability (≥.75) (2) |
| Beck Depression Inventory II (BDI II) (3) | Depressive symptoms of the past 2 weeks | Item 21 (interest in sexuality) was not examined in our study to adapt the BDI to the younger participants | Internal consistency (German version): α ≥ .84, retest reliability *r* ≥ .75 (4), application to adolescent sample validated(5) |
| Beck Anxiety Inventory  (BAI) (6) | Somatic anxiety symptoms of the last week |  | Internal consistency: α = .92 (7), Retest reliability (German version) *r* = .78 (8), acceptable psychometric properties in adolescent samples (9) |
| Sleeping Trait Questionnaire  (self-designed) | Chronotype, regularity of bedtimes, the duration of sleep needed to feel rested, and the frequency of sleeping through school or work |  | items used in the questionnaire are based on the Sleep Habit Survey (SHS)(10) the original SHS showed an acceptable internal consistency for the subscales with an α-value between 0.70 and 0.79 (10) (11) |
| Sleep Questionnaire B  (SF-B/R)(12) | Sleep quality of the last 2 weeks |  | Internal consistency of the factor scales: healthy sample: α= .47 - .87, psychiatric sample: α= .68 - .92, retest reliability *r*= .51 - .78, convergent validity: medium correlations with medical diagnoses referring to sleep, depression, and personality traits (12) |
| Stress and Coping Questionnaire for Children and Adolescents  (SSKJ 3-8 R) (13) | Behaviour in stressful situations and coping strategies |  | Internal consistency of the factor scales: α= .67 - .89, retest reliability *r*= .61 - .82 (13) |
| Pubertal Development Scale  (PDS) (14) | Self-reported current pubertal status |  | Internal consistency: α= .68-.83, correlation with interview ratings: *r*= .41 - .79 (15) |
| **Measurements during measurement week** | | | |
| **Daily measurements** | | | |
| Daily sleep questionnaire | satisfaction with the past night’s sleep, current mood and feeling of recreation, difficulties with sleeping in, nocturnal awakening, stress level of the past day, presence of nightmares, the consumed quantity of caffeinated beverages, the consumed quantity of alcoholic beverages as well as sleeping and wake-up time |  | See results for correlations with Sleep Questionnaire B |

**References**

1. Sheehan DV, Lecrubier Y, Sheehan KH, Amorim P, Janavs J, Weiller E, u. a. The Mini-International Neuropsychiatric Interview (MINI): the development and validation of a structured diagnostic psychiatric interview for DSM-IV and ICD-10. Journal of clinical psychiatry. 1998;59(20):22–33.

2. Sheehan DV, Sheehan KH, Shytle RD, Janavs J, Bannon Y, Rogers JE, u. a. Reliability and validity of the Mini International Neuropsychiatric Interview for Children and Adolescents (MINI-KID). J Clin Psychiatry. März 2010;71(3):313–26.

3. Hautzinger, M., Keller,F., Kühner,C. BDI-II: Beck Depressions-Inventar: Revision: Manual. Pearson Assessment; 2009.

4. Kühner C, Bürger C, Keller F, Hautzinger M. [Reliability and validity of the Revised Beck Depression Inventory (BDI-II). Results from German samples]. Nervenarzt. Juni 2007;78(6):651–6.

5. Dolle K, Schulte-Körne G, O’Leary AM, von Hofacker N, Izat Y, Allgaier AK. The Beck Depression Inventory-II in adolescent mental health patients: Cut-off scores for detecting depression and rating severity. Psychiatry Research. 30. Dezember 2012;200(2):843–8.

6. Margraf, J., Ehlers, A. Beck-Angst-Inventar [BAI]. Deutschsprachige Adaptation des Beck Anxiety Inventory von AT Beck und RA Stern. Frankfurt: Harcourt; 2007.

7. Beck AT, Epstein N, Brown G, Steer RA. An inventory for measuring clinical anxiety: Psychometric properties. Journal of Consulting and Clinical Psychology. 1988;56:893–7.

8. Geissner E, Huetteroth A. Beck Anxiety Inventory deutsch – Ein reliables, valides und praxisgeeignetes Instrument zur Messung klinischer Angst. Psychother Psychosom Med Psychol. März 2018;68(3/4):118–25.

9. Osman A, Hoffman J, Barrios FX, Kopper BA, Breitenstein JL, Hahn SK. Factor structure, reliability, and validity of the Beck Anxiety Inventory in adolescent psychiatric inpatients. Journal of Clinical Psychology. 2002;58(4):443–56.

10. Wolfson AR, Carskadon MA. Sleep Schedules and Daytime Functioning in Adolescents. Child Development. 1998;69(4):875–87.

11. Wolfson AR, Carskadon MA, Acebo C, Seifer R, Fallone G, Labyak SE, u. a. Evidence for the validity of a sleep habits survey for adolescents. Sleep. 15. März 2003;26(2):213–6.

12. Görtelmeyer, R. Schlaffragebogen A und B: SF-A/R und SF-B/R, 1st Auflage[Sleep Questionnaires A and B: SF-A/R and SF-B/R]. Hogrefe; 2011.

13. Lohaus, A., Eschenbeck, H., Kohlmann, C. W., Klein-Heßling, J. Fragebogen zur Erhebung von Stress und Stressbewältigung im Kindes- und Jugendalter (SSKJ 3-8) [Questionnaire for the Measurement Stress and Coping in Children and Adolescents (SSKJ 3-8)]. Hogrefe; 2006.

14. Watzlawik M. Die Erfassung des Pubertätsstatus anhand der Pubertal Development Scale. Diagnostica. Januar 2009;55(1):55–65.

15. Petersen AC, Crockett L, Richards M, Boxer A. A self-report measure of pubertal status: Reliability, validity, and initial norms. J Youth Adolescence. 1. April 1988;17(2):117–33.
