# Supplementary material for: Sleep quality and the cortisol and alpha-amylase awakening responses in adolescents with depressive disorders
Source: BJPsych Open. 2024 Aug 6;10(5):e140. doi: 10.1192/bjo.2024.730 (PMC11698168; doi:10.1192/bjo.2024.730)
Supplement: Krempel et al. supplementary material 2 — Krempel et al. supplementary material [file S2056472424007300sup002.docx]

**Suppl. Table 2**

| Overview of MEMS saliva sampling times across the whole sample. | | |
| --- | --- | --- |
| Day | 30-min interval | 15-min interval |
| 1 | 30:11 (03:42) | 18:01 (07:45) |
| 2 | 31:46 (06:50) | 17:38 (06:18) |
| 3 | 31:35 (07:34) | 17:06 (06:32) |
| *Note.* Many participants reported opening the sample container in advance for preparation. | | |
