# Supplementary material for: Sleep quality and the cortisol and alpha-amylase awakening responses in adolescents with depressive disorders
Source: BJPsych Open. 2024 Aug 6;10(5):e140. doi: 10.1192/bjo.2024.730 (PMC11698168; doi:10.1192/bjo.2024.730)
Supplement: Krempel et al. supplementary material 3 — Krempel et al. supplementary material [file S2056472424007300sup003.docx]

**Suppl. Table 3**

ANCOVA Cortisol and Alpha-Amylase

|  | *SS* | *df* | *MS* | *F* | *p* | *d* |
| --- | --- | --- | --- | --- | --- | --- |
| **ANCOVA CORT** |  |  |  |  |  |  |
| Time | 0.75 | 1.59 | 0.47 | 1.30 | .274 | .30 |
| Time*PDS | 2.87 | 1.59 | 1.81 | 4.99 | .014 | .58 |
| Time*group | 0.23 | 1.59 | 1.42 | 0.39 | .628 | .17 |
| Error | 34.52 | 95.31 | 0.36 |  |  |  |
| PDS | 0.21 | 1 | 0.21 | 0.14 | .710 | .09 |
| Group | 0.71 | 1 | 0.71 | 0.47 | .497 | .18 |
| **ANCOVA AMYL** |  |  |  |  |  |  |
| Time | 6.68 | 1. 78 | 3.75 | 1.93 | .150 | .36 |
| Time*PDS | 13.34 | 1. 78 | 7.48 | 3.86 | .029 | .51 |
| Time*group | 4.89 | 1. 78 | 2.74 | 1.41 | .248 | .31 |
| Error | 207.68 | 107.01 | 1.94 |  |  |  |
| PDS | 55.58 | 1 | 55.58 | 3.39 | .071 | .47 |
| Group | 1.53 | 1 | 1.54 | 0..93 | .761 | .09 |

SS-Type III Sum of Squares, df-Degrees of freedom, MS-Mean Square, d-Cohen’s d, CORT-Cortisol, AMYL-alpha-amylase, PDS-Pubertal development scale, BDI-Beck Depression Inventory II, BAI-Beck Anxiety Inventory, group-depression group / control group.
