# Supplementary material for: Sleep quality and the cortisol and alpha-amylase awakening responses in adolescents with depressive disorders
Source: BJPsych Open. 2024 Aug 6;10(5):e140. doi: 10.1192/bjo.2024.730 (PMC11698168; doi:10.1192/bjo.2024.730)
Supplement: Krempel et al. supplementary material 4 — Krempel et al. supplementary material [file S2056472424007300sup004.docx]

***Supplementary information***

Detailed alpha amylase assay analysis

The concentration of alpha-amylase in saliva was determined using an enzyme kinetic approach (1). Saliva samples were processed using a Genesis RSP8/150 liquid handling system (Tecan, Crailsheim, Germany) (1). Initially, saliva was diluted 1:625 with double-distilled water via the liquid handling system (1). Twenty microliters of the diluted saliva, along with a standard, were then dispensed into transparent 96-well microplates (Roth, Karlsruhe, Germany) (1). The standard solutions, with concentrations of 326, 163, 81.5, 40.75, 20.38, 10.19, and 5.01 U/l of alpha-amylase, were prepared from a 'Calibrator f.a.s.' solution (Roche Diagnostics, Mannheim, Germany) and bidest water as a zero standard (1).

Subsequently, 80 μL of substrate reagent (alpha-amylase EPS Sys; Roche Diagnostics, Mannheim, Germany) was added to each well using a multichannel pipette (1). The microplate, containing the sample and substrate, was incubated in a water bath at 37°C for 90 seconds to initiate the reaction (1). A first interference measurement was immediately taken at a wavelength of 405 nm using a standard ELISA reader (Anthos Labtech HT2, Anthos, Krefeld, Germany) (1). The plate was further incubated for 5 minutes at 37°C before a second measurement at 405 nm was conducted (1). Increases in absorbance were calculated for both unknown samples and standards (1).

The absorbance changes of diluted samples were then converted into alpha-amylase concentrations using linear regression analysis performed for each microplate (Graphpad Prism 4.0c for MacOSX, Graphpad Software, San Diego, CA) (1). The intra-assay and inter-assay variances were determined to be 2.9% and 6.1%, respectively, for alpha-amylase (1).

1. Rohleder N, Wolf JM, Maldonado EF, Kirschbaum C. The psychosocial stress-induced increase in salivary alpha-amylase is independent of saliva flow rate. Psychophysiology. 2006;43(6):645–52.
